# Cognitive enhancement and authenticity: moving beyond the Impasse

**DOI:** 10.1007/s11019-022-10075-2

**Published:** 2022-04-26

**Authors:** Emma C. Gordon

**Affiliations:** Department of Philosophy, University of Glasgow, G12 8QQ Glasgow, Scotland

## Abstract

In work on the ethics of cognitive enhancement use, there is a pervasive concern that such enhancement will—in some way—make us less authentic (e.g., Bublitz and Merkel 2009; Juth 2011). Attempts to clarify what this concern amounts to and how to respond to it often lead to debates on the nature of the “true self” (e.g., Maslen et al. 2014) and what constitutes “genuine human activity” (e.g., Kass 2003). This paper shows that a new and effective way to make progress on whether certain cases of cognitive enhancement problematically undermine authenticity is to make use of considerations from the separate debate on the nature of authentic *emotion*. Drawing in particular on Wasserman and Liao (2008), the present paper offers new conditions that can help us assess the impact of cognitive enhancements on authenticity.

## Introduction

Cognitive enhancements are widely understood as ways of “[amplifying or extending] core capacities of the mind through improvement or augmentation of internal or external information processing systems” (Bostrom and Sandberg, 2009) in such a way as to make us *better than well* 1. This broad category includes traditional interventions like education alongside emerging brain-computer interface technologies and possible future nootropics drugs that boost focus, memory, and cognitive processing to ever-greater extents^2^. Such interventions hold promise for everything from improving well-being to accelerating our discovery of cures for fatal diseases and existential threats. However, bioethicists working on human enhancement have also raised a range of serious concerns about enhancement technologies, ranging from claims that it will exacerbate inequality^3^ to questions about the ethical ramifications of using drugs to adjust our moral character^4^. In the present context, we will focus on one particularly vexing question about cognitive enhancement biotechnologies which has received quite a bit of recent attention^5^: call this the *authenticity question*:

### Cognitive Enhancement Authenticity Question

under what conditions, if any, does the use of cognitive enhancement problematically reduce or undermine the user’s *authenticity*?

Answering the authenticity question in satisfactorily would amount to an important breakthrough in the bioethics of enhancement, especially as the relevant technologies (e.g., nootropic drugs but also intelligence augmentation) are becoming increasingly more advanced^6^.

Although some progress has been made in the authenticity debate, it has been slow – and has arguably reached a kind of impasse – partly on account of the particular methodological approach that attempted answers tend to exhibit. The approach, in broad outline form, has been to respond the authenticity question by *first* taking a stand on the complex question of how to best characterise the nature of the “true self”, where a key contrast point has been the distinction between *essentialist* and *existentialist* approaches to the true self.^7^ A related approach by Kass (2003) asks not about the true self, as such, but rather poses an equally foundational philosophical question, which is the question of what makes any kind of activity genuinely *human* activity. Common to these approaches is a methodology that takes us deep into the philosophical weeds.

It will be shown that there is a promising alternative way to pursue the question which avoids the above theoretical morass, and a proof of concept of the efficacy of this alternative approach is found in the literature on *emotional authenticity.* In that literature, the starting point is slightly, but crucially, reframed – from a question about the *authentic self* to questions about authentic *attitudes* or appraisals – and from this more specific starting point, progress has been much more expedient. The aim in what follows will be to show that the strategy that has yielded fruit in debates about emotional authenticity can be equally effective in making progress on the Cognitive Enhancement Authenticity Question as it pertains to cognitive enhancement.

Here is the plan. § 2 briefly reviews the state of the literature on cognitive enhancement and authenticity. § 3 then canvasses some of the key moves in the parallel debate about emotional enhancement and authenticity, with special focus on a new strategy developed by Wasserman and Liao, which is used to characterize emotionally authentic attitudes. § 4 shows how we can reconceive the Cognitive Enhancement Authenticity Question in the cognitive domain in a way that permits us to envision a broadly analogous approach to characterizing in virtue of what cognitive attitudes are authentic when they are. A benefit of this strategy is that we can gain traction on Cognitive Enhancement Authenticity Question *without* taking any stand on either the nature of the self or about the nature of human activity as such.

## Existing Debates on Cognitive Enhancement Authenticity

The fact that the Cognitive Enhancement Authenticity Question has received attention among bioethicists of cognitive enhancement should not be surprising for several reasons. Firstly, authenticity is taken to be (in different ways) a valuable feature of a flourishing human life (see, e.g., Taylor 1992; Vannini and Williams 2016). Secondly, at least on the surface, it would seem that there is a kind of constitutive tension between authenticity and the use of enhancements aimed at *alter*ing ourselves by way of changing our dispositions, faculties, and attitudes (Juth 2011). In short: if we *change ourselves* with cognitive enhancement biotechnologies, might we not in doing so make ourselves less authentic, and in a way that undermines the value that authenticity contributes to our lives?

A natural starting point is to take a step back and ask first what the “true” self is (Bolt 2007), in order to get a contrastive grip on what features of oneself are *inauthentic* (with reference to which we might then assess whether features brought about by cognitive enhancements constitute *inauthentic* features). For example, in assessing whether deep brain stimulation might be a form of cognitive enhancement that would leave one inauthentic^8^, Pugh, Maslen and Savulescu (2017) maintain that as an “initial starting point, we can say that to be authentic is to live in accordance with one’s “true self” and from here reason that “The key [… ] is how we should identify those features of the self that are “true,” and those that are peripheral”; authenticity in action and thought is then understood (derivatively) in terms of actions and thoughts that in line with the true rather than peripheral features of the self (Bolt, 2007; Juengst and Mosely, 2015).

Two salient opposing characterisations of a “true self” are given by *essentialist* and *existentialist* views (see e.g., Erler and Hope 2015). *Essentialists* see the true self as some consistent, core part of ourselves—so, to discover the true self on the essentialist view is to perform a kind of voyage of self-discovery. From the essentialist starting point, as Maslen et al. (2014) point out, it might seem that we are most authentic when we are “natural” and unaltered. If so, using cognitive enhancement drugs and brain-computer interfaces to change our preexisting capacities and characteristics invariably makes us less authentic. In contrast, *existentialists* emphasise the importance to authenticity of *choosing* who to become, where choices manifesting authenticity are choices in alignment with values that we would endorse on reflection.^9^ From this starting point, it might seem that whether a given cognitive enhancement contributes to inauthenticity is to be settled by assessing to what extent their use reflects values that we would endorse (as opposed to, *per* essentialism, reflecting our core nature). In between essentialist and existentialist positions we can locate the *dual-basis* view (e.g., Levy 2011) and Pugh et al.’s (2017) coherentist view. Such approaches attempt to preserve the most plausible aspects of existentialism and essentialism—e.g., that there is some meaningful fact of the matter about our dispositions and that it is also possible for us to pursue meaningful change.

For our purposes, we need not take a stand on which of the above sorts of views best characterizes the true self. Rather, we want to emphasise two things. First, the task of characterizing the true self is a philosophically heavyweight task that itself opens further difficult questions about the “self”, about personal identity, and freedom.^10^ Given this, as Juengst and Mosely put it “no surprise that there is little consensus about whether enhancements undermine authenticity of the self” (Juengst and Mosely, 2015). Second, in the *context* in which the Cognitive Enhancement Authenticity Question is posed – a context that features opposing stances by bioconservative and bioprogressive bioethicists of cognitive enhancement – it will be difficult to defend a particular theory of the true self that does appear question-begging. Consider, for example, that essentialist views of the true self are naturally allied to bioconservatism, amd existentialist views with bioprogressivism (or transhumanism).

Against this background, it is well worth considering whether the (highly) relevant question of whether (and to what extent) cognitive enhancements undermine authenticity might be approached from a different angle.

## Emotional Authenticity and Enhancement

Questions about *emotional enhancement* and authenticity flow from similar concerns to those at the heart of debates about cognitive enhancement. For instance, if someone chooses to take a drug to induce feelings of happiness or grief, are their emotions resulting from the use of the drug less authentic?

In this literature, we find that a different strategy as the norm, one that reconceives the guiding question as a question about particular *emotions* rather than about the *self* as such.^11^ For example, Felicitas Kraemer (2011), in discussing emotional enhancement and authenticity, takes the target question of interest to be whether given particular *emotions* one feels as a result of the use of an enhancement are authentic emotions or inauthentic emotions. We can frame this *attitude-indexed* target question as follows:

### Emotional Enhancement Authenticity Question

for a given subject, S, an emotion E, and an emotional enhancement X, under what conditions does S’s use of X undermine the authenticity of E?

Whereas Kraemer ends up with a kind of subjectivist view of emotional authenticity which begins from this starting point, for our purposes, it will be more instructive to consider how Wasserman and Liao (2008) have proceeded from this same starting point – viz., by asking questions about the authenticity of emotions as opposed to questions about the true self.

As Wasserman and Liao (2008) see it, there are four plausible necessary conditions we can helpfully appeal to when assessing whether any given emotion is authentic: *responsiveness*, *non-exclusivity*, *proportionality*, and *lack of alienation*. Let’s briefly look at each of these conditions – conditions on the authenticity of *emotions*, rather than the authenticity of persons – and then in § 4 *cognitive analogues* of these four conditions will be described.

As a preliminary point, though, it will be useful to dispel an idea right out of the gates. When we think of authentic expressions of *persons*, it is common to point to spontaneity as a mark of authenticity (e.g., Larmore 1996; cf., Stevenson 2020). We might expect then that spontaneity would be a mark of an authentic *emotion*. On such a view, it would seem that drug-induced emotions might not be authentic *because* given the intervention of the drug, they would not be spontaneous. However, Wasserman and Liao reject this condition as too strong, as it would also rule out as authentic cases where we simply use internal methods of emotional regulation.^12^ For example, a spontaneity condition would suggest our affectionate emotions towards a partner are inauthentic if we re-arrived at them by reminding ourselves of the person’s good qualities after an argument. Indeed, even regulating our emotions through practices like meditation or mindfulness fails the spontaneity condition, so it seems wise to look elsewhere for appropriate constraints on authentic emotion.

Setting spontaneity aside, let’s look at the conditions they think are distinctive of authentic emotions, beginning with *responsiveness.* The *responsiveness* condition refers to the requirement that authentic emotions “typically” shift with the facts, becoming less intense or disappearing altogether when the relevant circumstances no longer obtain. For example, we typically stop feeling insulted, angry or hurt if we discover that our friend did *not* in fact insult us in the way we supposed. Wasserman and Liao’s core thought here is that if a drug prevented our happiness or our sadness from evolving in response to changes in circumstance, then what we’re feeling as a result of that drug is inauthentic.

Meanwhile, the *non-exclusivity* condition on emotional authenticity refers to our ability to have several salient emotions *competently*. So, for example, we can be joyful about securing a new job at the same time as being sad about our sibling’s recent breakup at more or less the same time. So, the idea goes, if an enhanced emotion is to count as authentic, it must be such that we could manifest other emotions at the same time (albeit with the disclaimer that authentic emotions can be “muted” by other authentic emotions).

The *proportionality* condition on authentic emotion tells us that authentic emotions are *appropriate to their object* and the capacity to fit their object, including by degree or intensity if compromised compromises the authenticity of an emotion. For example, we would not generally feel the same amount of grief at the death of a stranger as we would at the death of a loved one; shift the object of value (as experienced by the subject) and shift the degree of the felt emotion. By applying the proportionality condition, Wasserman and Liao want to mark as inauthentic drug-induced or drug-affected emotions that lack this nuance and are simply of equal intensity regardless of how we might vary the object.

Finally, there is the slightly more complex condition requiring *lack of alienation*. Here, Wasserman and Liao are reflecting on the possibility that emotional enhancement drugs could cause one to feel *alienated* from one’s previous self because of changes in responses induced by the drug that differ from one’s previous response patterns. For example, if you used to get angry easily and under the influence of the drug this is no longer the case (suppose you remain calm on an occasion when you’d previously snap), you might have a sense that you are no longer really “yourself” in some meaningful sense. Consequently, Wasserman and Liao believe that changes in emotionally dispositions must be *deliberately* sought for the related emotions to be authentic in a way that is not undermined by a sense of alienation.

Taken together these four conditions, note, are meant to be necessary conditions on the authenticity of a given emotion. An emotion can accordingly be less authentic by degree depending on whether one or more of these conditions are failed in a given case.

## Cognitive Enhancement and Authenticity: A New Strategy

We need not take a stand for the present purposes on whether the finer points of Wasserman and Liao’s response to the Emotional Enhancement Authenticity Question is correct.^13^ It might be, for instance, that slightly different necessary conditions are the correct ones, or that variations on the conditions they offer are more extensionally adequate.

Given that Wasserman and Liao’s methodological approach offers a way to theorise about authentic emotions *without* first getting bogged down in debates between essentialists and existentialists about the nature of the self or about the nature of human activity as such, it will be useful for our purposes to consider whether we might chart an analogous methodological path in the cognitive arena. A first step will be to revise the target question to a question about *beliefs* as opposed to a question about emotions^14^ (and, as opposed to – as per the original Cognitive Enhancement Authenticity Question – about *selves.*) What we get, accordingly, is the following analogical question:

### Cognitive Enhancement Authenticity Question-(Revised)

for a given subject, S, a belief, B, and a cognitive enhancement X, under what conditions does S’s use of X undermine the authenticity of B?

Interestingly, just as Wasserman and Liao did *not* include ‘lack of spontaneity’ among the conditions that would rule out an emotion as authentic, we also will not want any such condition on authentic *beliefs*, especially given that beliefs can vary -- significantly so – with respect to whether they are spontaneous or the results of careful reasoning. To help make this point concrete, consider Ernest Sosa’s (2015) distinction between *functional* and *judgemental* beliefs. An example of the former is the kind of action-guiding belief you regularly form in response to your immediate environment (e.g., ‘there is a table to my left’, or ‘I am approaching a wall’), but which is not such that its formation settles any particular inquiry into some ‘whether-p’ question you’ve undertaken. In the above examples, it is not as though these functional (in Sosa’s sense) beliefs constitute ‘answers’ to any questions you have. Compare now such beliefs with deliberate inquiries undertaken as to ‘whether p’ – e.g., whether a client is guilty, or whether a policy is fair. These inquiries may also terminate in beliefs, albeit *judgmental* beliefs – beliefs whereby one affirms in the endeavour to settle a question undertaken (Sosa 2015; 2021).

The functional/judgmental belief distinction helps us to sharpen why it would be a mistake to think that ‘lack of spontaneity’ is a condition we would want to appeal to help us rule out authentic beliefs. The problem, in short, is that such a condition would make our account of authentic beliefs too restrictive – such that, while functional beliefs would satisfy the condition, judgmental beliefs would fail it.

However, *responsiveness*, by contrast, *does* appear to be *prima facie* relevant to the authenticity of beliefs, albeit in a different sense than Wasserman and Liao maintain that it is relevant to authentic emotions. Whereas Wasserman and Liao hold that if a given emotion were prevented from evolving in response to changes in circumstance where there is a (normative) reason for the subject to feel differently, then what they are feeling is inauthentic. A variation on this idea is very plausible in the case of belief: to a first approximation, for a drug-derived belief to be authentic, it needs to be *reasons-responsive* in that one would typically ‘shed’ the belief (or at least be capable of doing so^15^) if circumstances no longer warrant it – viz., in the presence of *epistemic* reasons that count against the truth of the belief. To capture this idea more precisely, consider a version of this kind of proposal we find in the literature on freedom and moral responsibility, due to Fischer and Ravizza (1998). Their main concern is the question of when our beliefs are ‘free’ or autonomous in the way that is relevant to moral responsibility. The test they use to assess this is to ask whether you revise the belief (or not) in nearby worlds where you are presented with reasons to revise the belief.^16^ If the answer is ‘no’ (as it might be if the belief is, say, the result of brainwashing), then the belief fails their ‘freedom condition’ on moral responsibility.

Interestingly, note that the Fischer-Ravizza condition would also get the result that *enhanced* beliefs (say, e.g., those that are in some way the result of a cognitive enhancement) would fail to qualify as ones for which we bear responsibility *if* (holding fixed the enhancement that sustains the belief) it would not be revised in near-by worlds where we are presented with good reasons to believe otherwise. My proposed belief-based analogue of Wasserman and Liao’s responsiveness condition on authentic emotion can accordingly be appreciated kind of reasons-responsiveness condition the details of which can be unpacked modally (e.g., in terms of whether one sheds the belief in nearby worlds where one is presented with reasons to do so) in the broadly same way that Fischer and Ravizza unpack these details modally in their responsiveness condition on beliefs relevant for moral responsibility.^17^

And indeed, relying on this kind of condition gets us just the kind of result we should want – vis-à-vis authenticity – in cases of enhancement. If a drug caused one to perhaps ‘encode’ the belief so deeply that one wouldn’t easily shed it in the presence of clear counterevidence, it would with reference to the reasons-responsiveness condition we are envisaging not be authentic; this seems like just the right result. (Compare: *brainwashed* beliefs are paradigmatically inauthentic beliefs, and they clearly fail the reasons-responsiveness condition).^18^ However, on the other side of the coin, our modal reasons-responsiveness condition isn’t so strong that it rules out all cases of enhanced beliefs as inauthentic. Again, consider some kind of nootropic memory-boosting drug that helps individuals retain in memory beliefs they would otherwise have forgotten. The sustaining of such beliefs depends causally on the nootropic drug; however, it is *not* the case that such beliefs fail a reasons-responsiveness condition. A belief’s being sustained in memory in a way that relies on pharmacological cognitive enhancement is compatible with that belief’s being such that one would revise it in near-by worlds where they are presented with good reasons to do so.

What about *non-exclusivity?* It is slightly more complicated to see how to articulate a cognitive analogue of non-exclusivity (as a condition on authenticity) than it is in the case of reasons responsiveness. Recall that non-exclusivity, as a condition on authentic emotions, holds that the induction of the emotion by a given enhancement *should not prevent one from having other emotions*, even if they are temporarily muted or eclipsed by the presence of the relevant emotion induced. Of course, one has a myriad of beliefs, both occurrent and dispositional (Audi 1994); the idea of having an exclusive belief under any circumstances (enhanced or otherwise) is incoherent. This is particularly so when we consider the ubiquity of action-guiding functional beliefs in Sosa’s sense, as noted above.

*However*, we can begin to make sense of a kind of ‘non-exclusivity’ authenticity condition on beliefs when we consider the relationship between our *occurrent* beliefs (those whose content we are presently consciously considering and affirming) and our capacity for *attention*, viz., the selected ‘directedness’ of our mental lives.^19^

To a first approximation, we might think of an ‘non-exclusivity’ condition on beliefs constraining the exclusivity of the apportioning of our attention in a way that is at odds with the sense in which the relationship between our attention and our beliefs is ordinarily not so constrained. On this way of thinking, an enhanced belief (in order to be appropriately authentic) needs to *not* be such that it would *disproportionately* occupy one’s conscious attention. For example, if one is (as a result of the enhancement) *reflecting on* (at irrelevant times or disproportionately often) the *content* one’s enhanced beliefs, those beliefs are in this respect less authentic.

One might object to this characterisation by pointing out that in some cases, ‘myopic focus’ is compatible with a belief’s authenticity – viz., suppose, for instance, one has an important commitment the following day. It might be that one reflects consciously *on this fact* to the exclusion of other points of practical relevance throughout the day. As the objection goes, such myopic focus is not particularly out of the ordinary, especially in connection with events with important significance for one, and we aren’t included to take such focus as a marker of the inauthenticity of the belief on which one is focused. Such an account would be implausible restrictive.

While this objection might look initially problematic for the particular way of spelling out an analogous ‘non-exclusivity’ condition on authentic beliefs, it invites a straightforward response. Myopic focus and *disproportionate* focus in the sense that is relevant to the non-exclusivity condition come apart. The above example, for instance, features myopic focus but not disproportionate focus. The significance of a given event would plausibly warrant increased focus – and even if it did not generate a positive (normative) reason to apportion one’s attention this way – it would ordinarily serve as a good rationalising explanation for this kind of attention pattern. A plausible characterisation of the kind of non-exclusivity condition on authentic beliefs – framed in terms of attention – accordingly will be flexible enough to not rule out all beliefs that *merely* occupy more attention than other beliefs for a thinker. At minimum, such an account will need to make explicit that certain factors that contribute to the salience of a given belief (including practical factors) suffice to relax the threshold for which apportioning one’s attention to that belief at the exclusion of others qualifies as disproportionate.

By way of contrast, construing a *proportionality* condition on authentic beliefs is more straightforward than spelling out the non-exclusivity condition. Such a proportionality condition – modelled analogously off of a proportionality condition on authentic emotion – might hold, to a first approximation, that enhancement-derived beliefs must vary *in degree* when one acquires additional confirming or disconfirming evidence. The structural analogy with emotion here is as follows: just as it counts against the authenticity of an emotion (with reference to the proportionality condition) if the emotion cannot increase or decrease in intensity or severity in the presence of corresponding changes in perceived value/disvalue in the object of the relevant emotion, likewise, it counts against the authenticity of a belief (with reference to an analogous proportionality condition) if the relevant *credence* (viz., degree of belief) cannot increase or decrease in accordance with updated evidence.^20^ Put simply, a belief lacks authenticity if the credence representing the degree of belief is insensitive to normal mechanisms of updating in response to new evidence.

A critic might push back: wouldn’t this condition implausibly overgeneralise so as to rule beliefs for which one has maximally good evidence to be inauthentic, simply on the grounds that one is not in a position to update them? The answer here is, ‘no’. We can see why by drawing a comparison with theories of *sensitivity* in epistemology (e.g., Dretske 1971; Nozick 1981; cf., Pritchard 2008). On such theories, it is a necessary condition for a belief to qualify as knowledge that the belief is sensitive in the sense that, *were it false*, one would not to continue to believe it is true. Now, consider *necessary* truths – e.g., 1 + 2 = 2. Such truths, as the thought goes, couldn’t *possibly* be false. Does this mean that they are *not* thereby sensitive and thus (according to this theory) not known? ‘No’; the straightforward reason is that the counterfactual that characterises sensitivity (if it were false, I wouldn’t have believed it) is vacuously true rather than false; such beliefs, rather than being insensitive, are *trivially* sensitive. Something very similar is going on in the case of propositions for which we have maximally good evidence; our beliefs in such proposition (when based on this excellent evidence) needn’t be *insensitive* to good evidence against them simply on account of the fact that is no good evidence to be found against them. To think otherwise would be to construe the very idea of sensitivity in a way that is implausibly demanding (as would be illustrated in the example of necessary truths above).

Finally, we get to *lack of alienation*. Let’s clarify, firstly, the precise formulation of the condition in the emotional case, which takes into account Wasserman and Liao’s caveats in § 3. In particular, on their view, it counts against an emotion’s authenticity if the experience of the target emotion is accompanied by a sense of alienation with respect to one’s conception of one’s former self, *except* under circumstances under which the sense of alienation is an implication of a *deliberate* choice one made at a previous time to alter their emotional responses at a later time. Bearing in mind this caveat, we can canvass an initial articulation of an ‘anti-alienation’ condition (with an analogous caveat) in the cognitive case: to a first approximation, it counts against a *belief’s* authenticity if the experience of it is accompanied by a sense of alienation (with respect to one’s conception of one’s former self), *except* under circumstances under which the sense of alienation is an implication of a deliberate choice made at a previous time.

In practical terms, we might imagine here a case of cognitive enhancement via a Stentrode brain-computer interface, which is a brain implant that allows one to use thought commands to manipulate a computer.^21^ One might very well feel alienated *from* one’s previous self upon forming beliefs which (via the enhancement) have this kind of capacity to manipulate one’s environment. However, as the caveat on the condition holds, this fact alone needn’t count against the authenticity of the relevant beliefs (accompanied by this sense of alienation) *unless* it’s not the case that the difference in one’s present way of forming and exercising her beliefs is an implication of a deliberate choice made at a previous time. The situation is clearly different, however, if one’s forming and exercising her beliefs now is (even when accompanied by a sense of alienation) *not* a result of a deliberate prior choice (e.g., suppose, say, one is fitted with the Stentrode against her will, or in a clandestine way). Under this circumstance, the alienation experienced by forming and exercising these beliefs at a later time would count against the authenticity of those beliefs. A pleasing result of the above condition is that (as with our other conditions canvassed) the condition will rule out *some* cases of enhanced beliefs as inauthentic, but not all.

## Concluding Remarks

Taken together *responsiveness*, *non-exclusivity*, *proportionality*, and *lack of alienation* offer what look like necessary conditions on the authenticity of beliefs resulting from cognitive enhancement that are at least broadly as plausible as Wasserman and Liao’s conditions on the authenticity of emotions resulting from emotional enhancement.

Let’s consider this result now in the wider context in which we began in §§ 1–2. There we saw that central arena for adjudicating authenticity objections to cognitive enhancement was one in which the main parties to the dispute are taking stances on the *true self* (i.e., essentialist views, existentialist views, and hybrid views).

The approach suggested sidesteps debates about the nature of the true self; by shifting the target question to the Cognitive Enhancement Authenticity Question-(Revised), we investigate how to best spell out *necessary* conditions on a *belief’s* being authentic. Of course, one could spell such conditions out *with reference* to substantive theses about the nature of the true self. (For example, one with an antecedent commitment to an essentialist view of the true self might opt for a more stringent formulation of a ‘lack of alienation’ condition; one with an antecedent commitment to an existentialist view might be inclined to formulate such a condition in a more lax fashion). That said, in doing so one would need to answer questions about *selves* prior to answering questions about particular attitudes. As the track record of debate noted in § 2 indicates, it remains an advantage of the methodological option advanced here that one can gain traction on the authenticity of beliefs without *needing to first take any stance* on these heavyweight philosophical debates.

Going forward, then, we can see that there is – despite what the contemporary debate between essentialists and existentialists would suggest – a way past the current impasse. The more productive questions to be asking are questions about *which* are the best necessary conditions on *belief’s* authenticity, and how to best formulate them. The initial suggestions made in § 4 are, I hope, a promising start in this direction.

## Footnotes

1 On this widely accepted definition, enhancements are to be understood as distinct from therapeutic interventions (i.e., drugs and technologies that might help to address some form of pathology or dysfunction). See Juengst and Moseley (2015) for more on how we might define “enhancement.” For the purposes of this paper, however, the precise definition has little import, as the cases we will consider would plausibly count as enhancements on just about any view.

2 For an inventory of ethical issues that should guide the development and usage of future pharmacological cognitive enhancements, see Maslen et al. (2014). Meanwhile, see e.g., He et al. (2020) for a primer on brain-computer interfaces.

3 See e.g., Giublini and Minerva (2019) for an outline of the main positions on fair distribution of cognitive enhancements, along with their own proposal for how such enhancements could be used to improve equality of opportunity.

4 See Persson and Savulescu (e.g., 2012) for one of the central arguments in favor of moral enhancement. For a concise yet detailed summary of the key areas of disagreement in the human enhancement debate more broadly, see Juengst and Moseley (2016) and Clarke et al. (2016).

5 For work exploring the relationship between human enhancement and authenticity, see for example Pugh et al. (2017), Maslen et al. (2014), Bolt (2007) and Levy (2011).

6 For an introductory survey of new developments in brain-computer interfaces, see e.g., He at al. (2020). Meanwhile, see Zaami et al. (2020) for exploration of the latest and likely future uses of nootropic drugs.

7 These key difference between these two stances on the ‘true self’ is that essentialists take (in short) the true self as something to be discovered – viz., as determined by facts already in place about our nature – and existentialists take the self as something we are free to create through our choices in alignment with our values. These views are discussed in some more detail in § 2.

8 See Kraemer (2013) for discussion.

9 See here also Dworkin (1981), Frankfurt (1988), and Christman (1991; 2007).

10 See, e.g., Strohminger et al. (2017) and Sparby et al. (2019).

11 It is worth noting the parallel between this strategy and a similar strategy in debates about autonomy. On the one hand, a research question is whether and under which conditions a person *as such* is an autonomous person. On the other had separate question concerns *attitudinal autonomy* (e.g., Mele 2003) – namely, whether any given attitude one has is an autonomous attitude. One promising line of research conceives of the former kind of holistic autonomy *in terms* of one’s attitudinal autonomy; on this way of thinking, attitudinal autonomy is actually more fundamental than the autonomy of persons because the latter is explained in terms of the former. Plausibly, something similar might be said for authenticity; that is, we might separate questions of whether a person is authentic (holistic authenticity) from whether particular attitudes are authentic, and then conceive of the former in terms of the latter.

12 For an overview of empirical work in the psychology of emotional regulation, see Koole (2009).

13 Note that Wasserman and Liao take it that at least some drug-induced emotions do not plausibly meet these conditions, while others do.

14 It is worth registering that, at least on some strong cognitivist accounts of emotion (e.g. Nussbaum 2001; Solomon 1980; and Neu 2000) emotions are best understood as a species of belief. For example, on this picture, shame is just a matter of believing oneself to have failed to live up to a particular ideal. For an overview and critical discussion of this kind of position, see Scarantino (2010).

15 We needn’t assume here that subjects are perfectly responsive to objective epistemic reasons for belief.

16 The ‘near-by’ worlds is important here because, presumably, it is a triviality that any belief might be revised in the light of new evidence in *far-off* worlds – which is just another way of putting the controversially weak claim that all beliefs are *possibly* revisable in the sense of metaphysical possibility. What is of interest here intuitively is rather one would *likely* or easily enough revise the target belief in the face of new evidence – thus, we capture the ‘easily enough’ with reference to whether the belief is revised in specifically nearby worlds where one is presented with countervailing evidence.

17 For critical discussion of the Fischer-Ravizza approach in the case of moral responsibility, see, e.g., Mele (2006) and Bratman (2000).

18 See Mele (2003), and in particular, the discussion of brainwashing in connection with attitudinal autonomy.

19 I am remaining neutral for the present purposes of how to think about the substantive details of what directing one’s attention is best explained by and how it is related to consciousness (Schwitzgebel 2007). For discussion on both points, see e.g., Mole (2021).

20 See Sturgeon (2020) for an overview of the relationship between belief and credence.

21 For discussion, see https://www.medicaldevice-network.com/news/synchron-stentrode-trial-paralysis/.
